# Editorial: Cutaneous T-Cell Lymphomas 

**DOI:** 10.3389/fonc.2021.649714

**Published:** 2021-02-09

**Authors:** Catherine G. Chung, Basem M. William

**Affiliations:** ^1^ Departments of Pathology, The Ohio State University Wexner Medical Center and The James and Solove Cancer Hospital, Columbus, OH, United States; ^2^ Medicine, Division of Dermatology, The Ohio State University Wexner Medical Center and The James and Solove Cancer Hospital, Columbus, OH, United States; ^3^ Division of Hematology, The Ohio State University Wexner Medical Center and The James and Solove Cancer Hospital, Columbus, OH, United States

**Keywords:** cutaneous T cell lymphoma, brentuximab, PD-L1, mycosis fungoides, CAR T cell

Cutaneous T-cell lymphomas (CTCL), mycosis fungodies (MF) and its leukemic counterpart Sezary syndrome (SS), remain incurable malignancies with significant impact on patients’ quality of life (1). These are however exciting times for clinicians caring for patients with CTCL with approval of two highly effective agents in the past few years. Brentuximab vedotin (BV), a chimeric anti-CD30 monoclonal antibody conjugated to monmethyl auristatin E (MMAE) toxin, was approved by the US Food and Drug Administration (FDA) in 2017 based on the results of the phase III study (ALCANZA) that showed a superior overall response, lasting 4 months of 56.3% as compared to 12.5% for physician’s choice of methotrexate or bexarotene in patients with relapsed CTCL (2). Mogamulizumab, a humanized IgG1 anti-CCR4 monoclonal antibody, was also approved by the FDA in 2018, based on the phase III study (MAVORIC) that showed superior progression-free survival of mogamulizumab of 7.7 compared to 3.1 months with vorinostat in patients with relapsed CTCL (3).

In this Research Topic of *Frontiers in Oncology* focused on CTCL, Khan and Sawas review ongoing studies of antibody-directed therapies for MF/SS and the rationale for their use; among them are PD-1 and PD-L1 inhibitors nivolumab and pembrolizumab, both of which have had increasing applications in the treatment of various solid and hematologic malignancies. PD-L1 is expressed in a subset of patients with MF/SS, and pembrolizumab has shown a promising ORR of 38% in patients, in CITN-10 trial, with relapsed CTCL with a subset of patients experiencing very durable responses (4). Despite the emergence of multiple new targeted agents for the treatment of CTCL, skin-directed therapy remains the mainstay of treatment for early disease, and is reviewed in this issue by Tarabadkar and Shinohara, including current evidence and updated management recommendations of United States Cutaneous Lymphoma Consortium (USCLC).

The pathogenesis of CTCL remains incompletely understood and largely unknown. In this issue, several authors lend insights to our current understanding of lymphomagenesis in MF/SS. Ghazawi et al. reviews recent epidemiological data evaluating risk factors in the development of CTCL, including sex, age, race and various environmental, infectious, and iatrogenic exposures that shed light on possible mechanisms of malignancy in MF/SS. Gantchev et al. reports on the aberrant activation of meiosis genes in cells undergoing mitosis (a process termed “meiomitosis”) in the setting of CTCL, and demonstrate that there is differential gene expression of meiosis-specific cancer testis (meiCT) genes in a cohort of SS patients compared to healthy controls; these findings suggest that malignant cells in SS undergo meiomitosis, which may allow for the development of novel diagnostic tests that accurately distinguish CTCL from benign inflammatory conditions, as well as targeted therapies. Ferranti et al. discuss immunomodulation in CTCLs, and note that in a recent investigation of HIV-infected and non-HIV-infected patients with MF/SS, individuals with HIV demonstrated significantly higher survival and decreased risk of overall mortality compared to those without HIV. The authors also discuss exploiting retrovirus infection mechanisms for gene therapy in the treatment of CTCL, and review current studies to this end. In addition to identifying specific genetic aberrations in MF/SS involving TP53, the NFĸB pathway, and the JAK3/STAT3 signal transduction pathways that may provide specific targets for future therapies, these technologies have led to the identification of multiple distinct subpopulations with different drug sensitivities within a single patient (5), suggesting that the ideal treatment for CTCL may involve combination therapies informed by a patient’s unique malignant T-cell population. Phyo et al. summarizes new findings in the understanding of the biology of CTCL based on newer technologies that allow more precise molecular investigations of malignant T-cells including whole genome and whole exome sequencing and single cell RNA sequencing The evolving role of chimeric antigen receptor (CAR) T-cell therapy is summarized by Scarfo et al. highlighting the unique challenges in applying CAR-T cell therapy in the setting of T-cell malignancies including the consequences of T-cell aplasia and the killing of CAR-expressing cells by each other; a phenomenon described as “fratricide.”

This Research Topic of *Frontiers in Oncology* provides an overview of key issues relating to the pathobiology, current, and future, management of CTCL and should help identify areas of common interest between dermatologist, hematologists, and cutaneous biologists thus encouraging collaboration in both basic science research and translation into practice through national and international clinical trials. We hope you find this issue interesting and informative.

## Author Contributions

All authors listed have made a substantial, direct, and intellectual contribution to the work and approved it for publication.

## Conflict of Interest

The authors declare that the research was conducted in the absence of any commercial or financial relationships that could be construed as a potential conflict of interest.
